# Optimal Release Timing of Drain Clamping to Reduce Postoperative Bleeding after Total Knee Arthroplasty with Intraarticular Injection of Tranexamic Acid

**DOI:** 10.3390/medicina58091226

**Published:** 2022-09-05

**Authors:** Myung-Ku Kim, Sang-Hyun Ko, Yoon-Cheol Nam, Yoon-Sang Jeon, Dae-Gyu Kwon, Dong-Jin Ryu

**Affiliations:** Department of Orthopaedic Surgery, Inha University Hospital, Incheon 22332, Korea

**Keywords:** total knee arthroplasty, tranexamic acid, clamping time, transfusion, estimated blood loss

## Abstract

*Background and Objectives*: Intraarticular injection of tranexamic acid (IA-TXA) plus drain-clamping is a preferred method of reducing bleeding after total knee arthroplasty (TKA). However, no consensus has been reached regarding the timing of the clamping. The purpose of this study was to determine the optimum duration of drain-clamping after TKA with IA-TXA. *Materials and Methods*: We retrospectively reviewed 151 patients that underwent unilateral TKA with IA-TXA plus drain-clamping for 30 min, 2 h, or 3 h. The total drained volume was reviewed as the primary outcome, and hematocrit (Hct) reductions, estimated blood loss (EBL), transfusion rates, and wound complications were reviewed as secondary outcomes. *Results*: The mean total drained volume, Hct reduction, and EBL were significantly less in the 3 h group than in the 30 min group. Between the 2 h and 3 h groups, there was no statistical difference in the mean total drained volume, Hct reduction, or EBL. The proportion of patients who drained lesser than 300 mL was high in the 3 h group. No significant intergroup difference was observed for transfusion volume, transfusion rate, and wound related complications. *Conclusions*: In comparison of the IA-TXA plus drain-clamping after TKA, there was no difference in EBL between the 2 h group and the 3 h group, but the amount of drainage volume was small in the 3 h group.

## 1. Introduction

Total knee arthroplasty (TKA) is a major orthopedic surgical procedure used to treat end-stage osteoarthritic knees, and it has good clinical and functional outcomes [1]. However, TKA is associated with significant blood loss, and 18% to 67% of patients require a blood transfusion, which is associated with poor outcomes such as allergic reaction, extended hospitalization, thromboembolic events, and mortality [2,3,4]. For these reasons, various methods such as a tourniquet application, drain clamping, epinephrine or tranexamic acid (TXA), and fibrin sealant have been proposed to reduce blood loss [5,6,7,8,9,10,11].

TXA is a hemostatic substance that inhibits fibrinolysis, providing a pharmacological option to reduce blood loss. Previous studies have reported that intraarticular TXA (IA-TXA) combined with drain clamping is a more effective mean of preventing blood loss than drain clamping alone [12,13,14,15,16]. Liao et al. conducted a meta-analysis of the results of seven different studies and confirmed the efficacy of IA-TXA plus drain-clamping [16]. However, in this meta-analysis, little data were available for clamping times between 1 and 4 h, and it remains controversial (1 h: Onodera et al. [17], Mutsuzaki et al. [18]; 2 h: Sa-Ngasoongsong et al. [11,19]; 3 h: Chareancholvanich et al. [20]; 4 h: Wang et al. [21], Wu et al. [22]).

To the best of our knowledge, no study has compared blood loss with respect to drain-clamping time after TKA with IA-TXA. Accordingly, the present study was performed to determine an optimum drain-clamping time after TKA with IA-TXA by comparing blood loss and complication rates for different drain-clamping times. In this study, total drained volume was reviewed as the primary outcome and hematocrit (Hct) reductions, estimated blood loss (EBL), transfusion rates, and wound complications were reviewed as secondary outcomes.

## 2. Materials and Methods

This study was approved by our Institutional Review Board (IRB No. INHAUH 2020-03-035 at 20 April 2020). The requirement for informed consent was waived due to the retrospective nature of the study. The medical and surgical records of 151 patients that underwent TKA surgery at our hospital from January 2017 to December 2019 were retrospectively reviewed. According to our database, 328 patients underwent TKA surgery after being diagnosed with knee osteoarthritis. However, 177 patients were excluded after applying the following exclusion criteria: simultaneous bilateral TKA; concomitant operation; TKA with lateral retinacular release; TKA with patella resurfacing; use of an extended stem; a diagnosis of secondary osteoarthritis; a neurologic disorder; and the receipt of medications, such as antiplatelet or anticoagulant medications, likely to interfere with findings.

**Figure 1:**
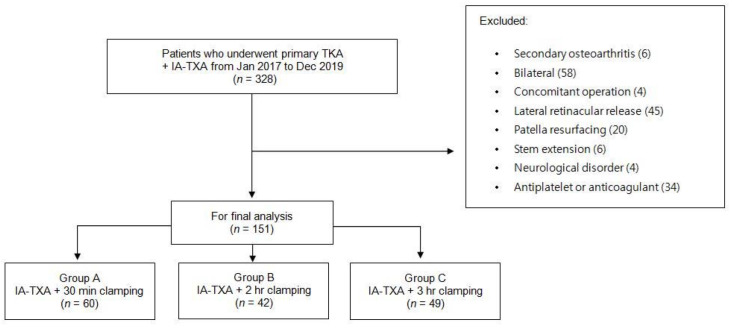
Flowchart of patient selection for this study.

The 151 study subjects were allocated to 3 groups according to clamping time; that is, Group A (*n* = 60) had a clamping time of 30 min, Group B (*n* = 42) had a clamping time of 2 h, and Group C (*n* = 49) had a clamping time of 3 h. A schematic of the patient selection is presented in Figure 1.

### 2.1. Operation Procedures

All patients underwent TKA by a single senior surgeon (MKK) and were provided with the same procedures and post-operative managements, except clamping time. Spinal anesthesia was used in most cases (93.4%), except for patients in whom spinal anesthesia was impossible due to severe degenerative deformation of the spine. In the supine position, a standard mid-line skin incision was made using a medial parapatellar approach after applying a pneumatic tourniquet at 350 mmHg. The same implant system (Persona-Zimmer Biomet, Warsaw, IN, USA) with cement fixation (Optipac 80, Biomet Orthopaedics GmbH, Dietikon, Switzerland) was applied to all patients, and a 3.2 mm drainage tube and a BAROVAC (400 mL, Sewoon Medical, Seoul, Republic of Korea, negative pressure 90 mmHg) comprised the drainage system. IA-TXA (3 g/30 cc + normal saline 70 cc) was administered immediately after joint capsule closure [23,24]; 1-0 Vicryl simple interrupted sutures were used for joint capsules and tendons, and 3-0 Vicryl simple running sutures were used for synovium. After subcutaneous and skin closures with 2-0 Vicryl and skin staples, respectively, we confirmed no leakage. An aseptic compression dressing was applied using an elastic bandage. Drains were clamped off in a timely manner.

### 2.2. Postoperative Management

All case-patients received the same perioperative management, including preemptive medications. Anti-embolism stockings and intermittent pneumatic compression were applied in all cases to prevent deep vein thrombosis (DVT) or pulmonary thromboembolism (PTE). In addition, anticoagulant (rivaroxaban, Xarelto^®^, 10 mg) was given from postoperative days (POD) 3 to 14. Patients with a hemoglobin level of <7 g/dL received a unit of packed red blood cells (RBCs), and patients that maintained a hemoglobin level between 7 and 9 g/dL received a unit of packed RBCs postoperatively [25]. All patients performed weight-bearing exercise (using a walker), active thigh lifting exercise, passive range of motion (ROM) exercise, and cryotherapy from POD 1 to 14 in consultant with the Department of Rehabilitation.

### 2.3. Perioperative Laboratory Factors and Hemodynamic Factors

Because of massive irrigation during TKA, blood loss under anesthesia cannot be measured appropriately. We used Nadler’s formula adjusted for height and body weight using Mercuriali’s formula to calculate blood volume from preoperative and POD 5 hematocrit values [26,27]. Mercuriali’s and Nadler’s formulae are as follows.

$$EBL=Blood\;volume\times \left(Hct_{preop}-Hct_{5\;days\;postoperative}\right)+volume\;of\;transfused\;RBCs\;Blood\;volume\;in\;men\;\left(L\right)=Height\;{\left(m\right)}^{3}\times 0.367+Body\;weight\;\left(kg\right)\times 0.032+0.604\;Blood\;volume\;in\;women\;\left(L\right)=Height\;{\left(m\right)}^{3}\times 0.356+Body\;weight\;\left(kg\right)\times 0.033+0.183$$

### 2.4. Outcomes Measurement

Total drained volume was reviewed as the primary outcome. Drainage amounts were recorded at 24 and 48 h postoperatively, and all drains were removed at 48 h postoperatively. For secondary outcomes, we measured Hct reduction and EBL with Hct value at pre-operation and POD 5. The transfusion rate was calculated by counting the number of patients who received a transfusion after surgery.

### 2.5. Complications

Complications such as DVT and PTE were evaluated closely because they can occur during clamping. From POD 2, surgical wounds were monitored to evaluate superficial infections and wound complications such as major bruises, oozing, hemarthrosis, subcutaneous hematoma, and blisters. Wounds were assessed up to POD 12 to evaluate possible superficial or deep wound infections. Major bruises were defined as bruises that extended >5 cm around wounds. Oozing was defined when three or more gauzes were soaked with blood.

### 2.6. Statistical Analysis

Results are presented as means ± standard deviations for continuous variables and as numbers and relative frequencies for categorical variables. Groups were compared using one-way analysis of variance (ANOVA) for quantitative data or Pearson’s chi-squared test for qualitative data. The test of significance was conducted on IBM SPSS Statistics for Windows version 25.0 (IBM Corp., Armonk, NY, USA), and statistical significance was accepted for *p*-values < 0.05. A post hoc power analysis was performed among the three groups using the G power 3.1 software (JMP., Lane Cove, NSW, Australia). The statistical power was 0.9505, which means that the number of subjects and the results were significant.

## 3. Results

### 3.1. Patient Demographics

Age, gender, surgery side, height, weight, blood volume, anesthesia method, and preoperative Hct level before surgery are presented in Table 1. No significant difference was found between these variables in the three groups (all *p* > 0.05).

### 3.2. Drainage Amount

**Figure 2:**
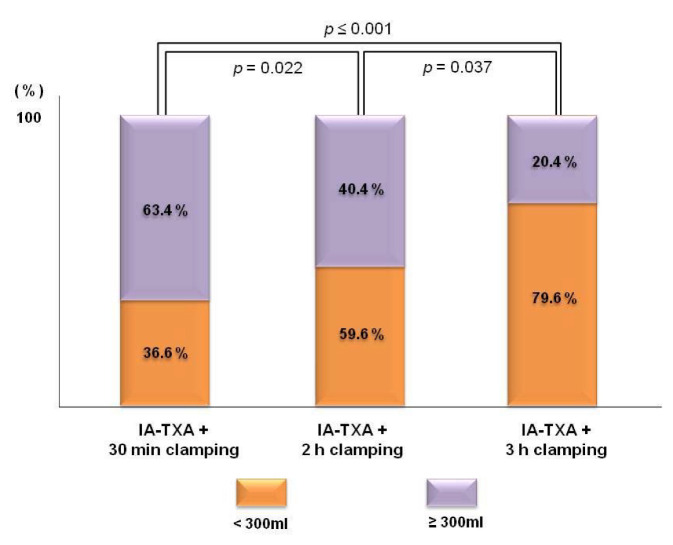
Percentages of patients with drainage volumes of <300 mL. Percentages of patients with a drainage volume of <300 mL increased with drainage time.

Mean total drainage amounts at 48 h postoperatively in groups A, B, and C were 332.3 ± 100.2, 286.4 ± 127.9, and 255.8 ± 84.5 mL, respectively (*p* = 0.001). Group C had a significantly lower amount than group A (*p* = 0.001), but no significant difference was observed between groups A and B (*p* = 0.09) or groups B and C (*p* = 0.495) (Table 2). The proportions of patients in the three groups with a drainage amount < 300 mL were 36.6%, 59.6%, and 79.6%, respectively. Notably, as clamping time increased, the percentage of patients with a drainage amount of <300 mL also increased (Figure 2).

### 3.3. Total Blood Loss

Mean EBL calculated using Mercuriali’s and Nadler’s formulae was higher in Group A than in the other groups (*p* ≤ 0.001) (Table 2). Mean EBL showed a decreasing tendency as clamping time increased.

### 3.4. Need for Transfusion

Regarding the need for transfusion, results showed a tendency similar to EBL and drainage amounts. Mean transfusion volume was highest in group A and tended to decrease with clamping time. Transfusion rates showed a similar tendency and were 16.7, 9.5, and 4.0% in groups A, B, and C, respectively.

### 3.5. Complications

No deep vein thrombosis or superficial infection occurred. Wound complications were categorized as major bruises, hemarthrosis, subcutaneous hematomas, and blisters, but no significant intergroup difference was observed (Table 3).

## 4. Discussion

Previous studies have reported that IA-TXA combined with drain clamping effectively prevents blood loss after TKA [12,13,14,15,16]. However, to our knowledge, no study has determined the optimum timing of drain clamp release. In the present study, TKA with IA-TXA plus drain clamping for 3 h resulted in a significant blood loss reduction compared to clamping for 30 min. Between the 2 h and 3 h groups, although there were no statistical differences, the proportion of patients who drained a lower volume than 300 mL was high in the 3 h group. No significant intergroup difference was observed for complication rates. Blood loss is an important postoperative consideration that must be considered after TKA. Bleeding into soft tissues surrounding the knee increases pain, stiffness, and length of recovery following surgery [28]. TXA application has recently become one of the most popular methods for reducing blood loss and transfusion requirements. TXA is an antifibrinolytic agent and was discovered in 1962. It prevents the formation of plasmin, inhibiting the breakdown of fibrin clots and decreasing bleeding. Although TXA has been administered intramuscularly, intravenously, and intraarticularly, it is being increasingly administered locally due to theoretically lower rates of systemic effects, including those related to thromboembolic disease [13,23,24,29]. However, due to safety concerns regarding PTE, interest is growing in the use of TXA as an IA agent in TKA. In the present study, we decided to use IA-TXA to reduce blood loss after TKA.

IA-TXA with drain-clamping reduces blood loss in TKA compared to IA-TXA without clamping [16]. This method is considered effective for reducing bleeding by forming a tamponade before opening. Prior studies have examined many methods, such as the intermittent method and specific timed drain clamping after surgery to reduce blood loss postoperatively [18,20,21,22]. However, no study has determined the optimum timing of drain clamp release after TKA with TXA. Liao et al. [16] conducted a systematic review and meta-analysis on the efficacy of TXA plus drain-clamping in TKA and concluded that this technique reduced blood loss and the need for transfusion. However, the seven clinical studies [11,17,18,19,20,21,22] included in their meta-analysis [16] were conducted using different clamping times (1 h: Onodera et al. [17], Mutsuzaki et al. [18]; 2 h: Sa-Ngasoongsong et al. [11,19]; 3 h: Chareancholvanich et al. [20]; 4 h: Wang et al. [21], Wu et al. [22]) and TXA dosages (range 250 to 1000 mg). To avoid confusion, we tried to define an effective clamping time by injecting TXA at 3 g/30 cc + 70 cc of normal saline and found EBL decreased and the percentage of patients with a drainage amount of <300 mL increased as the clamping time increased from 30 min to 3 h.

When clamping is released early, effective bleeding control cannot be achieved, i.e., longer clamping times are required to form effective tamponades. Since the half-life of TXA is 3 h, we examined the effects of clamping times up to 3 h [29]. Furthermore, it should be noted that complications such as hematoma can occur when clamping times are excessive (ca. > 4 h), as accumulations of blood in knee joints can lead to swelling, delayed wound healing, and increased risk of infection [14]. We found no significant difference between the three groups in terms of complications such as DVT, superficial infections, and wound complications.

The cytotoxic effect of IA-TXA on cartilage should be considered when the surgical intention is to preserve native cartilage tissues; its cytotoxicity may not affect total joint arthroplasties involving the removal of entire articular cartilage. Effective dosing for topical TXA ranges from 15 to 100 mg/mL. Increased exposure time to TXA at high concentrations is cytotoxic to cartilage. Because patients included in this study did not undergo patella resurfacing, we needed to minimize TXA exposure time and concentration on the articular surface and, thus, decided to use TXA at a concentration of 30 mg/mL and to limit the maximum exposure time to 3 h.

This study has several limitations. First, this is a retrospective study. Although we excluded the confounding factors that could affect bleeding tendency, a randomized control trial is needed for exact evaluation. Second, a relatively small number of cases were included in each group because all operations were performed by one surgeon in a single center. Third, postoperative blood loss was low in some patients when the surgeon was able to well identify and cauterize bleeding vessels, though vessel bleeding was carefully cauterized in all cases. Fourth, individual bleeding tendencies differ, and numerous factors can affect blood loss. However, considering the size of our cohort, we excluded factors that may have confounded results, such as a history of anticoagulant/antiplatelet medication, abnormal coagulation factors, and patients at a high risk of blood loss. Fifth, as blood loss could not be accurately measured, EBL was calculated using Mercuriali’s and Nadler’s formulae. However, Nadler’s formula calculates blood volume based on weight and height; thus, fluid-induced body changes pre-operation to POD 5 may have introduced errors. Sixth, postoperative pain is an important evaluation factor, and blood management can affect postoperative pain. However, in this study, we focused on blood loss rather than postoperative pain. Seventh, because of the limited sample size and low prevalence of perioperative complications, this study design was insufficient to draw a clear conclusion towards complications. Thus, we performed a power analysis among the three groups using the G power 3.1 software, and the power calculated was 97.9%. Despite these limitations, we believe our findings are meaningful as they provide evidence of the optimal duration of the drain-clamp application with IA-TXA after TKA. Further, our study provides orthopedic surgeons with a rationale for how to minimize bleeding after TKA.

## 5. Conclusions

Temporary drain clamping after TKA with an intraarticular injection of tranexamic acid can effectively reduce EBL. Although there were no statistical differences between the groups of 2 h and 3 h in terms of blood loss, the proportion of patients who drained lesser than 300 mL was notably higher in the 3 h group. In comparison to IA-TXA plus drain-clamping after TKA, there was no difference in EBL between the 2 h group and the 3 h group, but the amount of drainage was small in the 3 h group.

## Figures and Tables

**Table 1:** The patients’ preoperative characteristics data.

Characteristics	Time of Clamp Release			
	30 min (*n* = 60)	1 h (*n* = 42)	2 h (*n* = 49)	*p*-Value
Age at surgery (year) ^a^	70.9 ± 6.8	71.8 ± 7.9	69.9 ± 7.5	0.517
Gender ^b^				0.805
Female	47 (78.3%)	31 (73.8%)	36 (73.5%)	
Male	13 (21.7%)	11 (26.2%)	13 (26.5%)	
Side ^b^				
Right	33 (55.0%)	18 (42.9%)	26 (53.1%)	0.453
Left	27 (45.0%)	24 (57.1%)	23 (46.9%)	
Height (cm) ^a^	160.7 ± 4.7	161.6 ± 5.5	159.4 ± 8.2	0.429
Weight (kg) ^a^	62.2 ± 5.6	60.9 ± 7.8	60.5 ± 8.1	0.614
Blood volume (L) ^a^	3.78 ± 0.38	3.81 ± 0.54	3.74 ± 0.62	0.947
Anesthesia ^b^				0.666
General anesthesia	5 (8.3%)	3 (7.1%)	2 (4.1%)	
Spinal anesthesia	55 (91.7%)	39 (92.9%)	47 (95.9%)	
Preoperative Hct level (%) ^a^	37.3 ± 3.7	37.6 ± 3.0	38.5 ± 3.5	0.425

Hct: Hematocrit. ^a^ Data presented as mean ± standard deviation. ^b^ Data presented as number of patients having that condition (percentage of this group).

**Table 2:** Blood loss and blood transfusion outcome in three groups.

Variable	Time of Clamp Release			*p*-Value			
	30 min (*n* = 60)	2 h (*n* = 42)	3 h (*n* = 49)	Over-All Significance	30 min vs. 2 h	30 min vs. 3 h	2 h vs. 3 h
Drain amount (mL) ^a^							
24 h	240.2 ± 92.6	183.8 ± 96.9	143.2 ± 82.5	0.001	0.236	<0.001	0.185
48 h	130.1 ± 65.5	103.2 ± 65.5	81.3 ± 53.8	0.010	0.046	0.021	>0.999
Total	332.3 ± 100.2	286.4 ± 127.9	255.8 ± 84.5	0.001	0.09	0.001	0.495
Decreasing Hct (%) ^a^	10.8 ± 2.3	8.7 ± 2.0	6.6 ± 2.2	<0.001	<0.001	<0.001	<0.001
EBL (mL) ^a^	513.6 ± 276.3	396.7 ± 212.5	280.6 ± 182.0	<0.001	0.085	<0.001	0.112
Transfusion							
Transfusion volume (mL)	106.7 ± 253.7	66.7 ± 174.8	32.7 ± 137.5	0.146			
Transfusion rate ^b^	9 (16.7%)	4 (9.5%)	2 (4.1%)	0.165			

^a^ Data presented as mean ± standard deviation. ^b^ Data presented as number of patients having that condition (percentage of this group). Statistical significance was determined by one-way ANOVA followed by Scheffe’s post hoc analysis.

**Table 3:** Complications.

Variable	Time of Clamp Release			
	30 min (*n* = 60)	2 h (*n* = 42)	3 h (*n* = 49)	*p*-Value
Deep vein thrombosis	0 (0%)	0 (0%)	0 (0%)	0.999
Superficial infection	0 (0%)	0 (0%)	0 (0%)	0.999
Wound complications ^a^	6 (10.0%)	4 (9.5%)	5 (10.2%)	0.994
Major bruise	1 (1.7%)	2 (4.8%)	1 (2.0%)	0.600
Hemarthrosis	3 (5.0%)	2 (4.8%)	3 (6.1%)	0.951
Subcutaneous hematoma	1 (1.7%)	0 (0%)	1 (2.0%)	0.667
Blisters	1 (1.7%)	0 (0%)	1 (2.0%)	0.667

^a^ Data presented as number of patients having that condition (percentage of this group). Data presented as number (%). Statistical significance was determined by Pearson’s chi-squared test.

## Data Availability

Not applicable.
